# Incidental Perforation of Aortic Valve Leaflet Found on Presentation of Cardiogenic Shock

**DOI:** 10.7759/cureus.39476

**Published:** 2023-05-25

**Authors:** Austin Reed, Suhaib Bajwa, Shelby Schuh, Mary Mikhael

**Affiliations:** 1 Internal Medicine, University of Missouri, Columbia, USA

**Keywords:** acute aortic regurgitation, undifferentiated shock, aortic valve leaflet injury, cardiogenic shock, aortic valve disease

## Abstract

Aortic regurgitation (AR) is grouped into acute or chronic AR. Acute AR, unlike chronic AR, can manifest with significant hemodynamic compromise. Acute AR is typically due to endocarditis or aortic dissection, and less commonly due to blunt trauma or iatrogenic causes. We present a patient with cardiogenic shock due to severe acute AR from anterior leaflet perforation without an identifiable rheumatologic or infectious etiology.

## Introduction

Aortic regurgitation (AR) refers to the incomplete closure of the aortic valve leaflets resulting in regurgitant flow, which can be appreciated through the use of transthoracic echocardiogram in the parasternal long axis view with or without continuous flow doppler [1]. Measurement of velocity and pressure half time is important in quantifying the severe and guide acuity of management. Transthoracic echocardiograms can also be utilized to visualize the anatomy of the aortic leaflet, which is helpful in determining etiology. In chronic AR, the AR is typically more insidious in onset and is secondary to disorders affecting aortic valve leaflets rather than the enlargement of the aortic root. In terms of etiology for chronic AR, rheumatic heart disease remains the most common cause in the developing world, while aortic root dilation, congenital bicuspid aortic valve, or calcific disease remain the most common causes in the developed world [2]. In contrast to chronic AR, the most common causes of acute AR are endocarditis, aortic dissection, and on rare occasions, can include blunt trauma or iatrogenic damage [3]. Furthermore, acute AR, unlike chronic AR, can lead to significant hemodynamic abnormalities, including cardiogenic shock and pulmonary edema.

## Case presentation

A 44-year-old female with no past medical history presented to an outside hospital for 3 days of fever and arthralgias. She was started on ceftriaxone for treatment of a suspected urinary tract infection (UTI). However, despite the initiation of IV antibiotics, the patient continued to have persistent fevers and developed a new-onset headache. A computed tomography (CT) scan of the patient’s head was performed and was negative for acute pathology. Over the course of the next few days, the patient developed hemodynamic compromise including respiratory distress requiring intubation, and hypotension requiring the initiation of vasoactive medications. 

The patient was subsequently transferred to our facility with septic shock secondary to a UTI complicated by acute hypoxic respiratory failure. On arrival at our facility, the patient was intubated, mechanically ventilated, and afebrile with a temperature of 36.4 °C. She had a blood pressure of 104/59mmHg on 4mcg/min of norepinephrine, and a heart rate of 95 beats per minute. The patient was saturating 100% with a ventilator on pressure control mode with a positive end-expiratory pressure of 10, a fraction of inspired oxygen at 40%, and respiratory rates of 14 breaths per minute amounting to tidal volumes 400-500mL. Laboratory findings on admission are listed in Table 1. Blood cultures, fungal cultures, tracheal aspirate cultures, and a respiratory pathogen panel were unrevealing for a causative infectious organism. The urine culture from the outside hospital revealed *Streptococcus agalactiae* with 50,000-100,00 colony-forming units per mL (CFU/mL). Given the unclear source of infection, the antimicrobial regimen was broadened to vancomycin, ceftriaxone, acyclovir, and doxycycline for empiric coverage of possible meningitis and tick-borne diseases. A lumbar puncture and tick panel performed on the day of admission were unrevealing for acute infectious processes. 

**Table 1:** Pertinent laboratory findings on patient’s admissions WBC – White blood cell count. ESR – Erythrocyte Sedimentation Rate. CRP – C-Reactive Protein.

Laboratory Test	Result	Reference Range
WBC	22.61 x10^9^/L	3.5-10.5 x 10^9^/L
Hemoglobin	8.4 g/dL	13.5-17.5 g/dL
ESR	109 mm/Hr	0-30 mm/Hr
CRP	33.65 mg/dL	< 0.5 mg/dL

**Figure 1:**
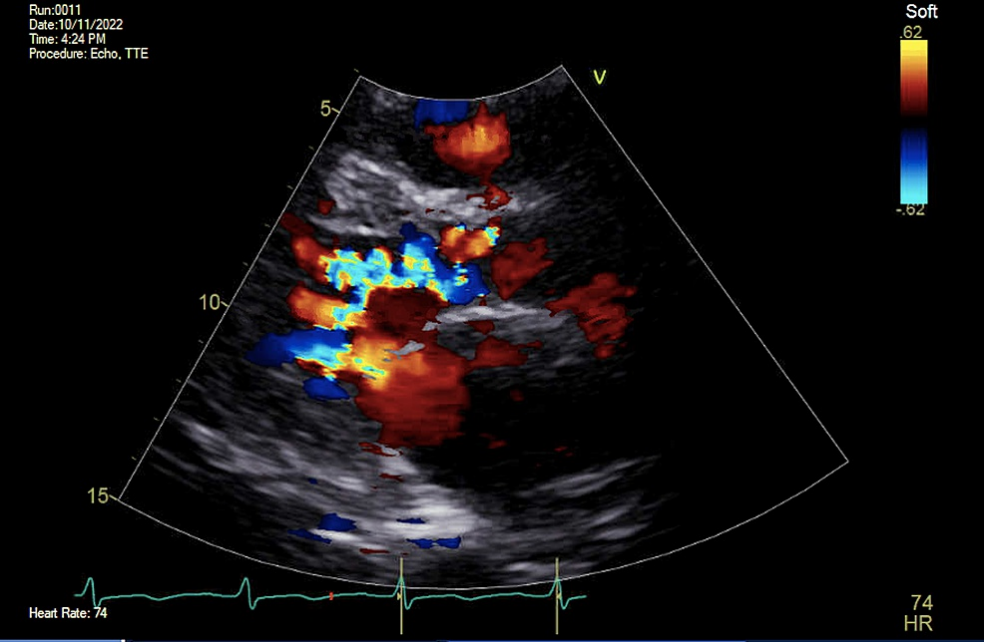
Transthoracic parasternal long echocardiogram view with Doppler demonstrating regurgitant flow from the aortic valve

**Figure 2:**
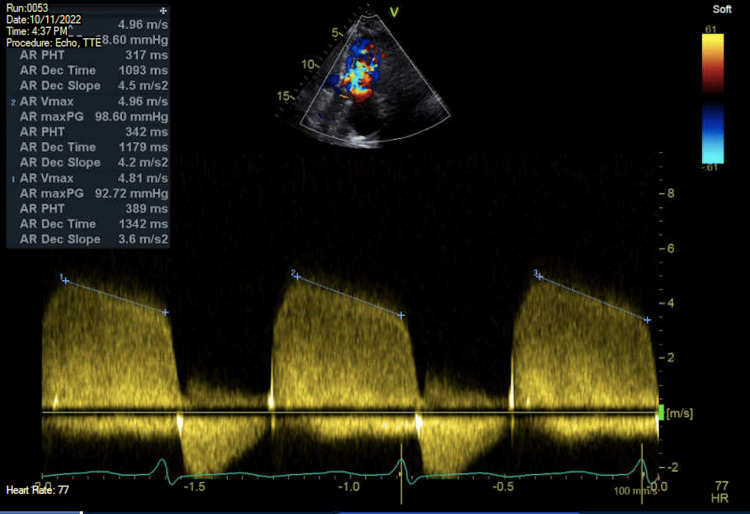
Two-chamber transthoracic echocardiogram view with Doppler evaluating regurgitant flow across the aortic valve

**Figure 3:**
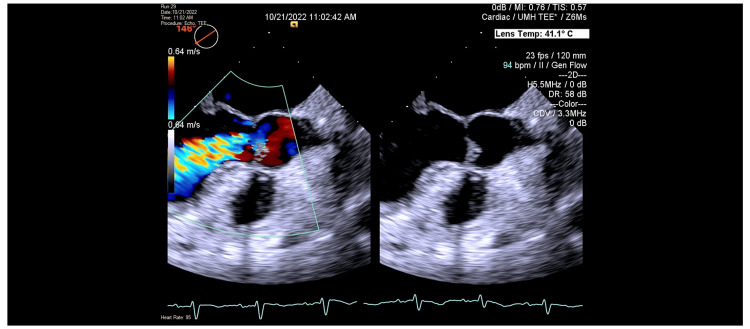
Long axis view of Transesophageal Echocardiogram demonstrating central jet regurgitant flow despite mobile anterior leaflet, suggestive of perforation.

On the second day of admission, a transthoracic echocardiogram (TTE) was completed for evaluation of other etiologies of shock, including cardiogenic shock. The TTE demonstrated normal left ventricular systolic function with an estimated ejection fraction of 65% with moderate aortic regurgitation, moderate to severe mitral regurgitation, and severely elevated pulmonary artery systolic pressures estimated at 60mmHg (Figures 1, 2). Diuresis with intravenous furosemide was initiated, and the patient’s hemodynamic and respiratory status improved with subsequent extubation and titration off of vasoactive medications on the third day of admission. Given the evidence of significant valvular abnormalities, a transesophageal echocardiogram (TEE) was performed which demonstrated severe aortic regurgitation with a central jet indicating likely perforation of the anterior leaflet (Figure 3). An extensive rheumatologic evaluation to differentiate the cause of leaflet perforation was negative, which included negative antinuclear antibody (ANA), negative rheumatoid factor (RF), negative anti-double-stranded DNA (anti-ds DNA), and negative cyclic citrullinated peptide (CCP). An additional Infectious workup was negative for human immunodeficiency virus (HIV), hepatitis C, hepatitis B, negative Herpes simplex virus (HSV), negative gonorrhea, and negative chlamydia. The Cardiology and Cardiothoracic surgery teams were consulted for the evaluation of the replacement of the aortic valve. Despite strong recommendations from numerous medical teams, the patient adamantly declined surgical intervention and was discharged home with diuretics and ongoing outpatient follow-up. 

## Discussion

The etiology of acute aortic regurgitation as determined from histologic analysis of surgically excised valves recorded in one retrospective study revealed that infective endocarditis accounted for 17% of cases, followed by aortic dissection at 10%. However, 34% of patients remained without a clear etiology of isolated AR based on histopathology [3]. Upon further literature review, there are a few reported cases of AR due to leaflet rupture following blunt trauma to the chest, with one specific example reported in a patient with Marfan Syndrome [4]. In this case, visualization of the valve leaflets excluded various etiologies. There was no evidence of vegetations present on the valve leaflets making infective endocarditis less likely. Additionally, the tri-leaflet anatomy was visualized, which excludes the possibility of bicuspid leaflet etiology. Lastly, the patient did not demonstrate marfanoid body habitus, did not report a family history of Marfan syndrome, and denied recent blunt trauma. 

Chronic AR progresses over months to years, allowing for the LV to remodel and dilate. These LV structural changes allow for the heart to accommodate an increased volume without any reflective increase in LV diastolic pressures, while simultaneously allowing for the heart to maintain adequate cardiac output. In the settings of acute AR, however, there is no time for the LV size to dilate or remodel, which results in elevated LV diastolic pressures, and a subsequent decrease in stroke volume and cardiac output. In acute AR, the heart attempts to compensate for the drop in stroke volume by increasing the heart rate, but in most cases, the overall cardiac output still decreases despite the reflexive increase in heart rate. This acute drop in cardiac output results in cardiogenic shock. Furthermore, pulmonary edema can develop because of an increase in diastolic volume in a normal-sized LV secondary to the regurgitant flow from the perforate aortic valve. The rapid increase in LV diastolic pressure results in both increased left atrial and pulmonary venous pressures, resulting in pulmonary edema [5]. 

While direct visualization of the aortic valve through TEE aided in the identification of spontaneous leaflet perforation, the patient’s presentation and hemodynamics suggested the timeline of development. The progression of chronic AR over months to years allows time for the left ventricle (LV) to remodel and dilate. This allows accommodation for increased volume without a reflective increase in LV diastolic pressures, while also maintaining adequate cardiac output. In acute AR, the LV is not given time to remodel or dilate, thus a decline in forward stroke volume occurs. Despite a reflexive increase in heart rate, the heart cannot meet the demands, and the cardiac output decreases. This acute decrease in cardiac output is known as cardiogenic shock, and on a physical exam, this can be seen as hypotension and tachycardia. Furthermore, pulmonary edema can develop because the increased volume from the regurgitant flow within a normal-sized LV causes a rapid increase in LV diastolic pressure that propagates to both increased left atrial and pulmonary venous pressures [5]. 

Although the hemodynamic findings of cardiogenic shock with rapid clinic decline are consistent with acute AR, the echocardiogram findings in this case demonstrated an LVEDV (Left Ventricle End Diastolic Volume) of 172 mL and LVESV (Left Ventricle End Systolic Volume) of 86.6mL. Both values are suggestive of chronic AR, as with chronic changes the LV has had time for remodeling. However, the right ventricular systolic pressures, a surrogate to LV diastolic and pulmonary venous pressure, were significantly elevated at 60 mmHg which is suggestive that the regurgitant flow from the perforated aortic valve led to elevated LV pressures, eventually resulting in pulmonary edema. These findings suggest that the patient had chronic AR, with an acute heart failure exacerbation resulting in cardiogenic shock. However, the exact timeline of the AV perforation remains unclear, thus not entirely eliminating a component of acute AR in this case. 

Surgery is imperative in the treatment of acute AR due to aortic dissection or infective endocarditis. However, medical therapy to reduce the LV afterload can assist with temporary stabilization [6]. Per the American Heart Association, the indications for aortic valve replacement for patients with chronic AR include the following: symptomatic severe AR, an asymptomatic patient with severe AR and evidence of decreased LV ejection fraction < 55%, or the need for concurrent cardiac surgery [6]. In this case, the utilization of intravenous diuresis allowed for LV afterload reduction and subsequent improvement in cardiovascular and respiratory function. Recommendations for surgical repair of this aortic valve given the likely acute perforation of the anterior leaflet were strongly emphasized by both the Cardiology and the Cardiothoracic surgical teams. This recommendation was extensively explained to the patient, however, the patient declined. Upon follow-up with the Cardiology services, the patient’s hypertension continued to be managed with chlorthalidone while ongoing consideration for surgical intervention occurred. 

## Conclusions

This case demonstrates a rare presentation of aortic valve perforation resulting in cardiogenic shock. Although rare, acute AR may result in catastrophic shock and pulmonary edema which can be masked in its discovery, leading to potentially fatal outcomes if not intervened upon early. Swift management with the use of bedside ultrasound in unstable patients is key to optimizing outcomes. Further workup for unclear etiology is also important, of which the differential diagnosis should remain broad. Treatment remains limited, but valve replacement therapy in the non-acute infectious periods may be reasonable and should be considered. 
